# *In vitro* Evaluation of ASCs and HUVECs Co-cultures in 3D Biodegradable Hydrogels on Neurite Outgrowth and Vascular Organization

**DOI:** 10.3389/fcell.2020.00489

**Published:** 2020-06-16

**Authors:** Luís A. Rocha, Eduardo D. Gomes, João L. Afonso, Sara Granja, Fatima Baltazar, Nuno A. Silva, Molly S. Shoichet, Rui A. Sousa, David A. Learmonth, Antonio J. Salgado

**Affiliations:** ^1^Life and Health Sciences Research Institute (ICVS), School of Medicine, University of Minho, Braga, Portugal; ^2^ICVS/3B’s – PT Government Associate Laboratory, Guimaraes, Portugal; ^3^Stemmatters, Biotecnologia e Medicina Regenerativa SA, Barco, Portugal; ^4^Institute of Biomaterials and Biomedical Engineering, University of Toronto, Toronto, ON, Canada

**Keywords:** vascularization, spinal cord injury, neurovascular, biomaterial, cell therapy, secretome, adipose-derived stem cells

## Abstract

Vascular disruption following spinal cord injury (SCI) decisively contributes to the poor functional recovery prognosis facing patients with the condition. Using a previously developed gellan gum hydrogel to which the adhesion motif GRGDS was grafted (GG-GRGDS), this work aimed to understand the ability of adipose-derived stem cells (ASCs) to impact vascular organization of human umbilical vein endothelial cells (HUVECs), and how this in turn affects neurite outgrowth of dorsal root ganglia (DRG) explants. Our data shows that culturing these cells together lead to a synergistic effect as showed by increased stimulation of neuritogenesis on DRG. Importantly, HUVECs were only able to assemble into vascular-like structures when cultured in the presence of ASCs, which shows the capacity of these cells in reorganizing the vascular milieu. Analysis of selected neuroregulatory molecules showed that the co-culture upregulated the secretion of several neurotrophic factors. On the other hand, ASCs, and ASCs + HUVECs presented a similar profile regarding the presence of angiotrophic molecules herein analyzed. Finally, the implantation of GG-GRGDS hydrogels encapsulating ASCs in the chick chorioallantoic membrane (CAM) lead to increases in vascular recruitment toward the hydrogels in comparison to GG-GRGDS alone. This indicates that the combination of ASCs with GG-GRGDS hydrogels could promote re-vascularization in trauma-related injuries in the central nervous system and thus control disease progression and induce functional recovery.

## Introduction

According to latest estimations, approximately 27 million people worldwide live with disabilities caused by spinal cord injury (SCI; James et al., 2019). This condition causes severe motor, autonomic, and sensory deficits. To date, treatment options are restricted to palliative care (Silva et al., 2014). Disruption of the vascular architecture of the spinal cord occurs concomitantly with injury and originates intraparenchymal hemorrhage, tissue edema, and swelling that leads to tissue ischemia (Mautes et al., 2000). Consequently, the blood spinal cord barrier (BSCB) is compromised and blood-borne molecules and inflammatory cells infiltrate the tissue indiscriminately (Bartanusz et al., 2011). Vascular damage initiates in gray matter and progressively extends into surrounding white matter leading to disrupted myelin and axonal and periaxonal swelling (Tator and Koyanagi, 1997). Altogether, these events exacerbate the already deleterious injury environment and contribute to the poor recovery scenario facing SCI patients. Endogenous attempts of revascularization (through angiogenesis) are observed from day 3 and peak about 1 week following injury, where some reports showed a return to basal vascular levels or even a 5-fold increase in vascular density at SCI injury site (Casella et al., 2002; Whetstone et al., 2003; Dray et al., 2009). This compensatory mechanism fails to integrate newly formed vessels into functional neurovascular units and most are pruned 2 weeks after injury. Additionally, Glut-1 transporters, which act as constant glucose transporters across the BSCB, are only reestablished at this time point and leave surviving neurons in a persistent metabolic imbalance (Whetstone et al., 2003). Recently it was shown that vascular perfusion below injury during the chronic phase of SCI was half that in comparison to normal spinal cords or above injury, resulting in local chronic hypoxia, and that transiently reestablishing oxygenation levels lead to brief motor recovery (Li et al., 2017). This finding highlights the relevance of vascularization therapies for SCI, showing that even though specific neuronal circuits bellow injury may remain functional, chronic hypoxia, and insufficient nutrient supply dictates their inability to undergo normal homeostasis. Thus, different approaches, either directly targeting vascularization (Rauch et al., 2009; Han et al., 2010; De Laporte et al., 2011; Yu et al., 2016) or not, concretely using neurotrophin-3-loaded chitosan (Duan et al., 2015), chitosan microhydrogels (Chedly et al., 2017), or a poly(lactic-co-glycolic) acid scaffold to deliver mesenchymal stem cells (MSCs; Ropper et al., 2017), revealed that modulation of this parameter is intricately involved in enhanced SCI recovery.

In this regard, transplantation of MSCs following SCI has shown protective effects to local vasculature (Matsushita et al., 2015; Morita et al., 2016; Vawda et al., 2019). Additionally, MSCs are also capable of promoting neuroprotection and modulation of the immune response toward a more regenerative-prone environment, which broadens the range of their effect and make them a promising candidate to treat SCI (Novikova et al., 2011; Spejo et al., 2013; Ribeiro et al., 2015). These effects have been extensively connected to the panel of molecules that MSCs secrete (secretome), including neurotrophic factors [brain derived nerve growth factor (BDNF), nerve growth factor (NGF), or glial-derived growth factor (GDNF)], pro-angiogenic molecules [vascular endothelial growth factor (VEGF), basic fibroblast growth factor (bFGF), or angiopoietin-1 (Ang-1)], or immunomodulatory molecules [monocyte chemoattractant protein-1 (MCP-1), transforming growth factor beta (TGF beta), or tumor necrosis factor alpha (TNF alpha)] though this profile varies across distinct MSC sources (Salgado et al., 2015; Pires et al., 2016). Among MSCs, adipose-derived stem cells (ASCs) are a clinically-relevant population for cell therapy applications as their isolation takes advantage of otherwise discarded tissue, being minimally invasive, and their transplantation does not elicit host immune response (Bunnell et al., 2008; Bronckaers et al., 2014). Despite the richness of ASCs’ secretome in angiogenic factors, different reports demonstrated limited capacity to produce fully branched vascular networks, contrasting to direct contact experiments where ASCs lead to the development of matured vascular structures, highlighting the advantage of including these cells in SCI therapies targeting vascularization (Merfeld-Clauss et al., 2010; Verseijden et al., 2010; Rohringer et al., 2014). Thus, including this type of MSCs in such therapies is appealing as it could possibly enable the modulation of vasculature toward homeostasis and overcome the host deficient response. Furthermore, endothelial cells positively affect neuronal proliferation and neurogenesis, being able to act as physical tracks for axonal growth (Li et al., 2013; Lange et al., 2016; Himmels et al., 2017; Paredes et al., 2018). The development of a cell therapy based on the transplantation of ASCs benefits from their capacity in protecting spared neurons, modulating the environment to a regenerative phenotype, whilst acting on the preservation of the BSCB and enhancing vascular organization following SCI. This can, in turn, contribute to quickly restore the compromised BSCB, controlling the infiltration of inflammatory cells and other inappropriate agents, preventing prolonged tissue hypoxia, providing simultaneously physical cues for neuronal regeneration.

To improve poor survival rates associated with cell transplantation, hydrogels are being used as they enable replication of the physical properties of the native central nervous system (CNS), whilst providing appropriate cues for cell survival, proliferation, and integration into host tissue (Orive et al., 2009; Khaing et al., 2014). Taking this into consideration, in this work we intended to develop a co-culture system based on the encapsulation of ASCs and human umbilical vein endothelial cells (HUVECs) within an in-house developed gellan gum (GG) matrix modified with the adhesion motif GRGDS (Silva et al., 2012; Gomes et al., 2016). This GG-based biomaterial has been previously reported as suitable to culture distinct neuronal cells and ASCs, which later translated into improved functional outcomes following its implantation in a SCI animal model.

The main goals of the present work were to study the simultaneous impact of ASCs in the vascular organization of HUVECs and neurite extension of dorsal root ganglia (DRG) explants, as well as to understand how the angiogenic and neuroregulatory nature of their secretome is altered by the presence of HUVECs in 3D conditions. Finally, we assessed the capacity of ASCs encapsulated in GG-GRGDS hydrogels to recruit blood vessels in a simple *in vivo setting* using the chick chorioallantoic membrane (CAM) assay.

## Materials and Methods

### Coupling of Maleimide-GRGDS to Furan-Gellan Gum

The coupling of GRGDS to GG was done using a two-step methodology where first GG is modified with furan by creating an amide bond, through the activation of its -COOH groups, and then coupled to maleimide-modified GRGDS (mal-GRGDS) taking advantage of Diels-Alder cyclization chemistry between the maleimide group of the peptide and the furan group of GG in accordance to previously described protocols (Silva et al., 2012; Gomes et al., 2016). A 1% (w/V) gellan gum (GG, Sigma, United States) solution was dissolved in 100 mM 2-(N-morpholino)ethanesulfonic acid (MES, Sigma, United States) buffer at pH 5.5 and 37°C. Then, a 750 mM 4-(4,6-Dimethoxy-1,3,5-triazin-2-yl)-4-methylmorpholinium chloride (DMT-MM, Sigma, United States) solution is added in a 1:4 molar ratio (GG:DMT-MM) to activate the-COOH groups of the polymer, which is followed by the addition of furfurylamine (Acros Organics, Belgium) using the same molar ratio. The reaction continues for 24 h and afterwards the obtained products are dialyzed in membranes with a cutoff of 12–14 kDa (Spectrum Labs, United States) to purify the modified polymer from reaction by-products alternatively against distilled water and PBS (0.1 M, pH 7.2) for 5 days. GG-furan was recovered as a white powder by removing its aqueous content by lyophilization. To immobilize the peptide in furan-GG, 1.2 mg/mL of the modified polymer was dissolved in 100 mM MES buffer at pH 5.5 and 37°C. After complete dissolution, mal-GRGDS peptide (Anaspec, United States) was added in a 1:5 molar ration (furan:maleimide), and the reaction continued under vigorous stirring for 48 h. The purification of the peptide-modified GG is done by dialysis (Mw cutoff 12–14 kDa) against distilled water and PBS (0.1 M, pH 7.2) in alternance. Removal of water by lyophilization allowed to obtain GRGDS-modified GG (GG-GRGDS) as a white powder. To quantify the amount of peptide immobilized onto the backbone of GG we performed an amino acid analysis. The protocol consists on the acidic hydrolysis of the peptide with 6 N HCl for 24 h followed by derivatization with phenylisothiocyanate. HPLC was used to quantify the derivatized hydrolizates. A defined amount of mal-GRGDS previously incubated with native GG suffered the same derivatization protocol and amino acid analysis and was used as a control.

### Cell Isolation and Culture

Adipose-derived stem cells were isolated by LaCell LLC from the lipoaspirates of consenting donors according to Dubois and coworkers (Dubois et al., 2008) under a protocol previously approved by an institutional review panel at LaCell LLC. Upon isolation, ASCs were cultured in α-MEM (Invitrogen, United States) supplemented with 10% Fetal Bovine Serum (FBS, Biochrom AG, Germany), and 1% (V/V) penicillin-streptomycin (pen/strep, Invitrogen, United States) at 37°C and 5% CO_2_ (V/V) with medium exchanges every 3 days.

Human umbilical vein endothelial cells were obtained from the umbilical cord of healthy consenting patients from the Gynecology and Obstetrics Service of Hospital de Braga using a protocol approved by the review board of the Ethical Commission for Health of Braga Hospital (CESHB). After rinsing and cleaning the umbilical cord with PBS, a cannula was inserted into the umbilical vein. Then, the vein was washed with PBS to remove blood clots and excesses of blood. Afterwards, the other extremity of the umbilical cord was closed with forceps and the vein was filled with α-MEM containing 0.2% (w/V; 210 U/mL) Type I Collagenase (Gibco, Thermo Fischer Scientific, United States) and 1% pen/strep. To allow for digestion, the umbilical cord was transferred into a cell culture incubator [*T* = 37°C and 5% (V/V) CO_2_] for 15 min. Before opening, the cord was massaged to guarantee a homogenous digestion and then its content transferred to a 50 mL Falcon, being subsequently washed with α-MEM having 10% FBS (w/V) and 1% pen/strep, PBS, and finally with a syringe filled with air. This was followed by the centrifugation of the suspension for 10 min at 1200 rpm, removal of the supernatant and resuspension of the pellet in Endothelial Growth Media (EGM, R&D Systems, United States) supplemented with 1× Endothelial Growth Supplement (EGS, R&D Systems, United States), and 1% (V/V) pen/strep. The cellular suspension was then equally divided into the wells of a 6-well plate pre-coated with 1% (w/V) Type B bovine gelatin (Sigma, United States) and cultured in Endothelial Growth Media (EGM, R&D Systems, United States) supplemented with 1× Endothelial Growth Supplement (EGS, R&D Systems, United States) and 1% (V/V) pen/strep at 37°C and 5% (V/V) CO_2_ overnight to allow the attachment of HUVECs. The following day media was changed to remove unattached cells and debris and from this point onwards media is exchanged every two days to keep purifying the culture. Upon confluence, part of the cells were stored in liquid nitrogen until further use and the rest were transferred to a T75 flask pre-coated with gelatin and cultured as previously described.

### Hydrogel Preparation

Lyophilized GG-GRGDS and unmodified GG was exposed to UV lights for 15 min (Silva et al., 2013). To produce hydrogels for the 3D environment experiments, a 1% (w/V) solution composed of equal parts of GG-GRGDS and unmodified GG was prepared and dissolved at 40°C in ultra-pure water. Prior to the experiments the polymeric solution was ionically crosslinked by adding 10% (V/V) of a 0.3% (w/V) CaCl_2_ [to a final concentration of 0.03% (w/V)]. The volume of hydrogels for the experiments was 50 μL.

### 3D Cell Cultures – ASCs, HUVECs and Their Co-Culture

Prior to their encapsulation in GG-GRGDS ASCs and HUVECs were cultured as detailed in section “Cell isolation and culture” and the hydrogels prepared according to section “Hydrogel preparation.” The pellets with the appropriate number of ASCs and HUVECs were resuspended homogenously in the corresponding volume of GG-GRGDS at a cell density of 30,000 cells/50 μL of hydrogel and cultured under previously described conditions for each cell type. The encapsulation of cells for co-culture experiments was done in a 1:1 ASCs:HUVECs ratio by mixing the appropriate volume of each cell suspension obtained subsequently to individual 2D cultures which was followed by centrifugation at 1200 rpm for 5 min to obtain the cell mixture pellet. The appropriate volume of GG-RGDS was then added to the pellet allowing formation of hydrogels with the previously referred density (15,000 ASCs + 15,000 HUVECs/50 μL of hydrogel) being cultured using α-MEM with 10% (V/V) FBS and 1% (V/V) pen/strep.

### Dorsal Root Ganglia (DRG) Isolation and Culture

Dorsal root ganglia explants were used to understand the capacity that co-culturing ASCs and HUVECs in GG-GRGDS has in inducing neurite outgrowth from the explants in comparison to each cell type cultured alone and GG-GRGDS without cells. Furthermore, using the same experimental setting this organotypic model allowed to assess the modulation of genes related to axonal growth and cytoskeleton dynamics (GAP43 and β-Tubulin III, respectively) along their time in culture. The isolation of DRG explants was effectuated using a previously detailed protocol (Gomes et al., 2016, 2018). Thus, DRG from the thoracic regions of the spine of neonatal pups (P5-7) were removed and placed in cold 1 × HBSS without Ca^2+^ and Mg^2+^ (Invitrogen, United States) with 1% (V/V) pen/strep. The remains of peripheral nerve processes were properly cleaned from DRG and then the explants were placed on top of the hydrogels across the 4 groups (no cells, ASCs, HUVECs, ASCs + HUVECs). The cell culture continued for 7 days in Neurobasal medium supplemented with 1 × B27 (Invitrogen, United States), 2 mM L-glutamine (Invitrogen, United States), 6 mg/mL D-glucose (Sigma, United States), and 1% (V/V) pen/strep with medium changes every two days and under a humidified atmosphere [37°C and 5% (V/V) CO_2_] before fixating the samples using PFA and performing immunocytochemistry (ICC) to understand neurite outgrowth as well as the morphology of ASCs and HUVECs inside the hydrogels.

Dorsal root ganglia collection for PCR analysis followed the same extraction and culture methodology and was done at multiple timepoints: 12 h, 24 h, 1 day, 4 days, and 7 days following culture in the hydrogels referring to the 4 s. Pools of 2 DRG were collected at each timepoint to eppendorfs containing TripleXtractor (Grisp, Portugal; a phenol and guanidine isothiocyanate-based solution to extract high quality RNA) and then subjected to RNA isolation or in alternative were rapidly frozen at −80°C until further use.

Cell encapsulation was performed 24 h before DRG culture and followed the methodology detailed in section “3D cell cultures – ASCs, HUVECs and their co-culture.”

### Immunocytochemistry (ICC) and Phalloidin/DAPI Staining

After 7 days of culture hydrogels and explants were fixed in 4% paraformaldehyde (PFA, Panreac, Spain) for 45 min at room temperature (RT). This step was followed by washing the samples 3 times with PBS and by permeabilizing cell membranes with 0.3% (V/V) Triton X-100 (Sigma, United States) for 10 min. To block non-specific binding sites, samples were incubated in PBS with 10% (V/V) fetal calf serum (FCS, Biochrom AG, Germany) for 1 h 30 min. Primary antibodies were then properly diluted in PBS 10% FCS and added to the samples for 48 h at 4°C. Mouse anti-neurofilament 200 kDa antibody (1:200, Millipore, United States) was used to unveil neurites and rabbit anti-CD 31 (1:20, Abcam, United Kingdom) to identify HUVECs. Following 3 washes using PBS with 0.5% (V/V) FCS, Alexa Fluor 488 goat anti-rabbit (1:1000, Invitrogen, United States) and Alexa Fluor 647 goat anti-mouse (1:1000, Invitrogen, United States) were diluted in PBS and added to the hydrogels overnight at 4°C. Following 3 washes with PBS, a PBS solution with 1 μg/mL of DAPI (Invitrogen, United States), and 0.1 μg/mL (Sigma, United States) was added to the hydrogels for 45 min at RT. Imaging was performed on a confocal point-scanning microscope Olympus FV1000.

### Neurite Extension and Outgrowth Analysis

The area occupied by the neurites of each DRG explant was calculated using the ImageJ (NIH) plugin Neurite-J (Torres-Espín et al., 2014) and using a previously developed protocol (Gomes et al., 2018). Therefore, after defining the scale, the area referring to the body of the DRG was defined and the threshold contrast properly corrected to emphasize its neurites. The image is automatically translated to 8 bits and using the function “Analyze particles” the area corresponding to the extension the neurites is calculated. The longest neurite was also quantified using Neurite-J after identifying again the DRG body the plugin automatically creates concentric rings with 25 μm intervals and is defining as the length at which the last ring is capable of intersecting neurites.

### Analysis of the Vascular Organization of HUVECs in GG-GRGDS

To analyze the vascular arrangement of HUVECs encapsulated in GG-GRGDS either in the presence or not of ASCs, AngioTool64 Version 0.6a was used (Zudaire et al., 2011). After opening the images referring to the fluorescence channel utilized for CD31 and defining the scale, the background and small particles were removed by defining the appropriate signal threshold in the software. After this correction the software automatically quantifies different parameters related to vascular organization such as total vessel length, vessel area, vessel percentage area, and number of junctions.

### RNA Extraction and qRT-PCR Analysis

Total RNA was extracted from pools of 2 DRG using TripleXtractor and following the instructions provided by the manufacturers. After quantifying the RNA using a NanoDrop 1000 spectrophotometer (ThermoFisher Scientific, United States) the samples were diluted to approximately 1 μg/μL and 1 μg of sample transcribed into cDNA using the Xpert cDNA Synthesis Mastermix (Grisp, Portugal) to the manufacturer’s protocol. Primers were designed using the Primer-BLAST tool (NCBI, United States) and the name of the genes, GenBank accession numbers and sequences are found on Table 1. The qRT-PCR reactions were done in a CFX96 real-time instrument (BioRad, United States) with the XPert Fast SYBR. mastermix and using equal cDNA concentrations for each sample following the manufacturer’s instructions. The expression levels of target genes (GAP-43 and β-Tubulin III) were normalized against housekeeping genes (GAPDH and HPRT-1) and presented as fold-change mRNA levels in comparison to the control group. The fold-change levels were calculated using the 2^ΔΔ*CT*^ method.

**TABLE 1:** Forward and reverse sequences of the primers used for qRT-PCR analysis and respective GenBank accession number, gene symbol, name, and product size.

**GenBank accession number**	**Gene symbol**	**Gene name**	**Primer sequence (5′→3′)**	**Size (bp)**
NM_017195.3	GAP43	Growth Associated Protein 43	**Fw:** CAA GCT GAG GAG GAG AAA GAA GC **Rv:** GCA GGA GAG ACA GGG TTC AGG T	158
NM_139254.2	Tubb3	Tubulin beta III	**Fw:** AGA CCC CAG CGG CAA CTA TGT **Rv:** CCA GCA CCA CTC TGA CCG AA	204
NM_017008.4	GAPDH	Glyceraldehyde 3-phosphate dehydrogenase	**Fw:** CAG TGC CAG CCT CGT CTC AT **Rv:** TGG TGA TGG GTT TCC CGT TGA	247
NM_012583.2	HPRT1	hypoxanthine phosphoribosyltransferase 1	**Fw:** CCT CAG TCC CAG CGT CGT GAT TA **Rv:** TCC AGC AGG TCA GCA AAG AAC T	231

### Secretome Collection From 3D Cultures

The collection of the secretome from 3D cell culture sections was performed after culturing and maintaining cells across the 3 conditions (ASCs, HUVECs and their co-culture) for 6 days as detailed in section “3D cell cultures – ASCs, HUVECs and their co-culture.” Subsequently, the hydrogels were washed 3 times with PBS and Neurobasal with 1% (V/V) pen/strep. This is followed by their incubation with Neurobasal with 1% (V/V) pen/strep during 24 h after which their secretome is collected, centrifuged at 1200 rpm for 5 min and the supernatant recovered and stored at −80°C until further use.

### Neurotrophic and Angiogenic Profile of 3D Secretomes

The evaluation of the angiogenic and neurotrophic profile of the previously obtained secretomes was performed using the Human Neuro Discovery Array C1 and Human Angiogenesis Array C1 (RayBiotech, United States) following the manufacturer’s guidelines. Briefly, each membrane was blocked for unspecific interactions using blocking buffer for 30 min at RT which was followed by its removal and incubation with 1 mL of secretome overnight at 4°C. Afterwards, the secretome was aspirated and the membranes washed using the washing buffers provided by the kit. Subsequently, 1 mL of biotinylated antibody cocktail was pipetted into each membrane and incubated for 2 h at RT. The antibody cocktail was removed, and the membranes washed using the same washing protocol. Then, 2 mL of 1 × HRP-Streptavidin was added to each well and incubated for 2 h at RT. The membranes were once again washed and prior to their revealing, 500 μL of a 1:1 mixture containing Detection buffer C and D was added for 2 min at RT. Finally, the chemiluminescence image of each membrane was obtained using a Sapphire Biomolecular Imager (Azure Biosystems, United States). The intensity of each dot was quantified using the AzureSpot software (Azure Biosystems, United States) by designing an 8 × 8 dot grid adjusted to include each individual point. Absolute values were normalized for the mean of the positive control of each membrane and the background subtracted to allow the comparison between membranes and secretomes.

### Chick Chorioallantoic Membrane (CAM) Assay

This simple *in vivo* system was used to evaluate the chemotactic capacity of GG-GRGDS hydrogels encapsulating ASCs in recruiting blood vessels toward the hydrogel in comparison to GG-GRGDS alone and collagen. The protocol was initiated by incubating white fertilized chicken eggs at 37°C and under a 40% humidified atmosphere for 3 days. After carefully cleaning the eggs with chlorohexidine and putting the egg racks in a laminar-flow hood a small hole in the smallest extremity of the egg was made 2 mL of albumin removed with a 20 G needle in a syringe to dissociate the CAM from the egg shell. Embryo viability was assessed after creating a circular window (approximately 3 cm) that allowed to check it and granted access to the CAM. Then, the opening was sealed with parafilm and the remaining eggs allowed to return to the incubated where they stayed for 1 week. At this point, GG-GRGDS hydrogels encapsulating ASCs, GG-GRGDS alone, and collagen were transferred to a zone with no major vascularization on top of a CAM, following opening the eggs and checking for their viability. GG-GRGDS hydrogels were produced 24 h before implantation as detailed in section “Hydrogel preparation” and ASCs encapsulated according to section “3D cell cultures – ASCs, HUVECs and their co-culture.” GG-GRGDS hydrogels with no cells were incubated in α-MEM supplemented with 10% (w/V) FBS and 1% pen/strep. Collagen hydrogels were done at the same time by mixing rat tail Collagen Type I [3.61 mg/mL, 89.6% (V/V), BD Biosciences, United States] with 10% (V/V) of 10× DMEM concentrated medium (Invitrogen, United States) and 0.4% (V/V) of a 7.5% (w/V) NaHCO_3_ solution. 50 μL hydrogel drops were then made and incubated for 2 h at 37°C and 5% CO_2_ (Gomes et al., 2018) for polymerization to occur. Then, the collagen hydrogels were incubated in α-MEM supplemented with 10% (w/V) FBS and 1% pen/strep until CAM implantation. Following 3 days of implantation the hydrogels were photographed *in ovo* using a stereomicroscope (Olympus S2x16) and the embryos sacrificed at −80°C for 10 min and fixated in 4% PFA at RT. The CAM portion harboring each hydrogel was dissected and excised using small scissors and transferred to 6-well plates. *Ex ovo* images of each CAM were taken and the total number of vessels directly converging to the hydrogels quantified using ImageJ which allowed to discriminate differences in this parameter between experimental groups.

### Statistical Analysis

Statistical analysis was performed using GraphPad Prism version 7.04 for Windows (GraphPad Software). Neurite outgrowth as well as CAM experiments were analyzed by performing one-way ANOVA followed by the Bonferroni *post-hoc* test. Welch’s *t*-test allowed to assess differences among groups for the vascular arrangement experiments and two-way ANOVA with Tukey’s multiple comparisons test to assess differences between groups at each timepoint. Differences were considered statistically significant if a *p*-value ≤ 0.05 was observed (95 % confidence level).

## Results

### Successful GRGDS Engraftment in GG

**FIGURE 1:**
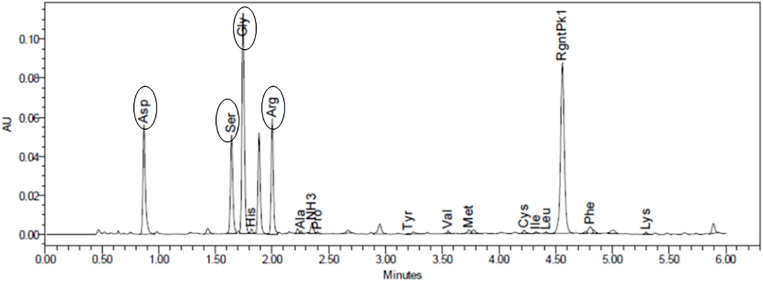
Amino acid analysis allowed to quantify the amount of peptide bound to gellan (92.85 nmol of GRGDS/mg of gellan). Oval forms identify each peptide.

The modification of GG followed a click chemistry approach previously optimized and published by our group (Silva et al., 2012; Gomes et al., 2016). Quantification of the total peptide bound to GG using HPLC-based amino acid analysis showed 92.85 nmol of GRGDS per mg of GG (Figure 1).

### The Presence of ASCs Is Fundamental to Increase Neurite Outgrowth on DRG Explants and the Vascular Assembly of HUVECs

**FIGURE 2:**
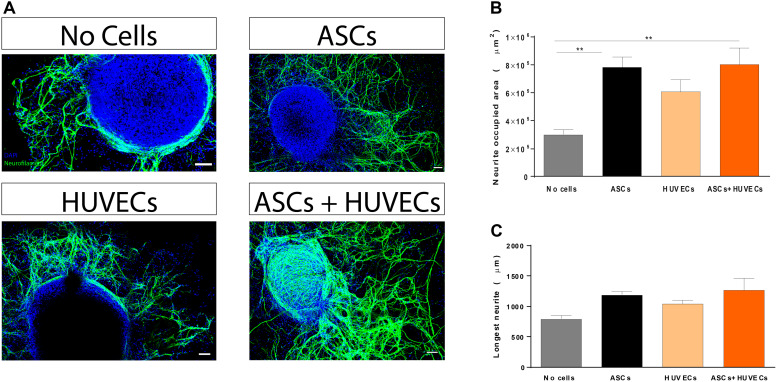
Effect of co-culturing ASCs and HUVECs on GRGDS-modified gellan gum in the neurite outgrowth of DRG explants in comparison to each cell type alone. **(A)** Representative images of the conditions. **(B)** Co-culture promoted a similar outgrowth to ASCs, being statistically significantly higher than the hydrogel without cells. **(C)** The longest neurite followed the same trend but without statistical differences. Scale bar: 100 μm. Values are shown as mean ± SEM (*n* = 8/10); **p < 0.01.

Given the lack of self-regenerative capacity observed in SCI, understanding how this parameter is affected is of pivotal importance during the initial steps of the development of a therapeutic approach to treat the condition (biomaterial-based or not). Thus, modulation of neuritogenesis by co-culturing ASCs and HUVECs in GG-GRGDS was compared to each cell type alone and the hydrogel by itself using a DRG organotypic model for axonal regeneration. Following 7 days of culture it was observed that both ASCs and the co-culture promoted increased neurite outgrowth (Figure 2A). Quantification of the area occupied by neurites provided similar values for GRGDS-GG encapsulating ASCs and the co-culture (7.83 ± 0.75 × 10^5^ μm^2^ and 8.0 ± 1.2 × 10^5^ μm^2^, respectively), being superior to HUVECs alone (608757 ± 0.86 × 10^5^ μm^2^), and statistically significantly higher (*p* < 0.01) than GG-GRGDS (2.99 ± 0.35 × 10^5^ μm^2^; Figure 2B).

**FIGURE 3:**
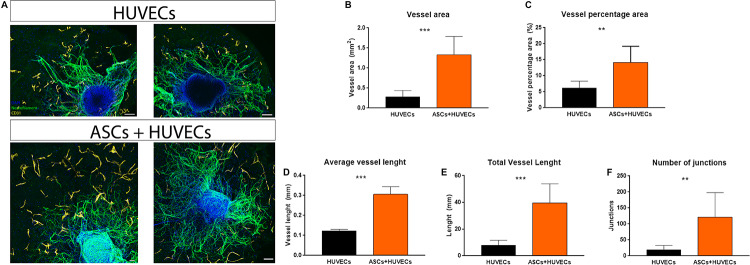
**(A)** Vascular organization of HUVECs inside GG-GRGDS when cultured alone and in co-culture with ASCs. These MSCs promoted the organization of the endothelial cells in vascular-like structures with a statistically significant increase on **(B)** vessel area, **(C)** vessel percentage area, **(D)** average vessel length, **(E)** total vessel length, and **(F)** number of junctions. Scale bar: 100 μm. Data is shown as mean± SEM with *n* = 8/10. **p < 0.01, ***p < 0.001.

A biomaterial-based strategy that aims to promote a revascularization therapy for SCI must provide furnish adequate conditions for ECs to assemble into vascular structures. Therefore, this was another parameter analyzed during these experiments. When encapsulated alone in GG-GRGDS hydrogels, HUVECs were found to be interspersed along the hydrogel with no obvious assembly into vascular-like structures. This was in complete contrast to what occurred when co-culturing these ECs with ASCs, where the vascular organization of HUVECs was noticeable (Figure 3A). The presence of ASCs statistically significantly increased several parameters related to vasculature such as vessel area (0.28 ± 0.07 mm vs 1.32 ± 0.16 mm; *p* < 0.001), vessel percentage area (6.1 ± 0.90% vs 14.16 ± 1.78%; *p* < 0.01), average vessel length (0.12 ± 0.08 mm vs 0.31 ± 0.07 mm; *p* < 0.001), total vessel length (7.79 ± 1.58 mm vs 39.72 ± 5.02 mm; *p* < 0.001), and the number of junctions formed by the vascular bed (19.50 ± 5.39 mm vs 121.30 ± 27.10 mm; *p* < 0.01; Figures 3B–F). These results show that ASCs have the capacity to induce vascular re-organization of biomaterials and how this modulation can be important to create a positive interplay on ECs and finally impact axonal growth.

### GAP-43 Expression Rapidly Increases in DRG From GG-GRGDS Encapsulating ASCs and Co-Culture Groups as ß-Tubulin III Decreases

**FIGURE 4:**
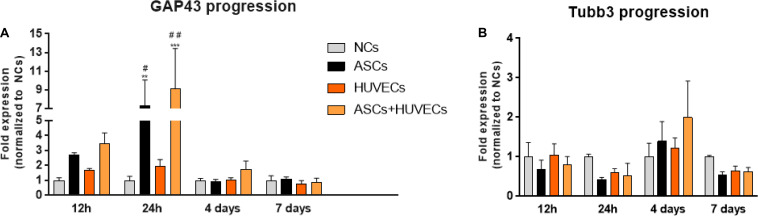
Temporal dynamics of the expression of GAP-43 and βIII-tubulin in DRG cultured on GG-GRGDS encapsulating ASCs, HUVECs, ASCs+HUVECs, or no cells. **(A)** The co-culture condition increases the expression of GAP43 as early as 12 h in comparison to all the other conditions, reaching the maximum at 24 h (statistically significant to no cells and HUVECs), and decreasing at latter timepoints (4 and 7 days). **(B)** The expression of βIII-tubulin follows an opposite dynamic and shows that the induction of plasticity is dependent on the downregulation of cytoskeleton genes. Scale bar: 100 μm. Results are shown as mean ± SEM (*n* = 4, each represents a pool of 2 DRG). **p < 0.01 and ***p < 0.001 in comparison to no cells; ^#^*p* < 0.05 and ^##^*p* < 0.01 in comparison to HUVECs.

The temporal dynamics of the genetic expression of GAP-43 (highly expressed in the growth cone of regenerating neurons) and ß-Tubulin III (Tubb3, neuronal cytoskeleton) allowed to understand how the use of ASCs, HUVECs and their co-culture could be modulating neurite extension from DRG in comparison to the hydrogel without cells. Analyzing Figure 4A it is possible to conclude that ASCs and the co-culture upregulated the expression of GAP-43 in DRG neurons as early as 12 h following culture (2.71 ± 0.14 fold for ASCs and 3.47 ± 0.71 fold for ASCs + HUVECs), reaching its maximum at 24 h (ASCs: 5.53 ± 2.8 fold, ASCs + HUVECs: 9.1 ± 4.33 fold) where statistically significant differences were found to HUVECs (*p* < 0.05 for ASCs and *p* < 0.01 for ASCs + HUVECs) and GG-GRGDS alone (*p* < 0.01 for ASCs and *p* < 0.001 for ASCs + HUVECs) during the same timepoint. The genetic expression of this axonal growth-related protein markedly decreased at the 4 days timepoint for both conditions where ASCs presented a lesser decrease following 7 days of culture.

GAP-43 expression in DRG cultured together with HUVECs encapsulated in GG-GRGDS was increased at the 12 and 24 h timepoints relative to the hydrogel alone (1.71 ± 0.10 and 1.94 ± 0.46 fold, respectively) but without the dramatic increase detailed for the other two conditions. Interestingly, the expression of Tubb3 followed the opposite path (Figure 4B). Thus, the expression of this neuronal cytoskeleton gene was downregulated for ASCs and ASCs + HUVECs at 12 h (ASCs: 0.69 ± 0.23 fold; ASCs + HUVECs: 0.81 ± 0.20 fold), and 24 h of culture (ASCs: 0.42 ± 0.06 fold; ASCs + HUVECs: 0.53 ± 0.31 fold). This gene, however, was slightly upregulated at 4 days of culture for both conditions (1.39 ± 0.50 fold for ASCs and 2.00 ± 0.92 fold for ASCs + HUVECs) which was followed by its downregulation at 7 days of culture (ASCs: 0.54 ± 0.09 fold; ASCs + HUVECs: 0.62 ± 0.11 fold). The expression of Tubb3 for DRG cultured with HUVECs presented a more homogeneous dynamic (1.046 ± 0.29 fold at 12 h; 0.60 ± 0.10 fold at 24 h; 1.23 ± 0.25 fold at 4 days; and 0.646 ± 0.12 fold at 7 days). These results show that the combination of GG-GRGDS hydrogels with ASCs or ASCS+HUVECs was able to stimulate axonal growth, which was reflected by increased GAP-43 levels at 12 h and 24 h following culture, contrarily to HUVECs where upregulation of this gene was not as markedly as seen for the other two conditions. On the other hand, it seemed that for neurite outgrowth to occur the cytoskeleton of DRG neurons had to be disturbed. These results are in line with what was observed in the neurite outgrowth experiments described in Figure 2.

### Neurotrophic and Angiogenic Signature of the Secretome of ASCs + HUVECs Is Distinct From Each Cell Type Alone

The results obtained on DRG experiments showed a clear beneficial effect on HUVECs by the presence of ASCs, with enhancement on their organization into vascular-like structures. This was followed by an increased capacity of the co-culture and ASCs in promoting neurite outgrowth from the explants in relation to HUVECs and prompted the study of the neurotrophic and angiogenic character of their secretomes. The full array of biomolecules detected, and their relative expression can be found in Supplementary Figure 1.

**FIGURE 5:**
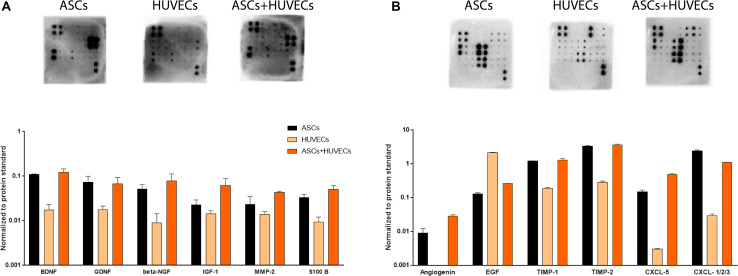
Analysis of the secretomes of ASCs, HUVECs, and their co-culture after 7 days of culture in GG-GRGDS allowed to understand the relative expression of a panel of neuroregulatory and angiotrophic molecules. **(A)** The secretome of ASCs+HUVECs showed an upregulation on different neurotrophic factors (BDNF, β-NGF, IGF-1, and S-100 B) showing a positive effect of the interaction of both cells on the secretion of these molecules. **(B)** The secretome of HUVECs presented decreased amounts of the quantified angiogenic molecules, with exception to EGF, whereas ASCs+HUVECs upregulated Angiogenin and CXCL-5 and ASCs promoted increased expression of CXCL-1/2/3.

Starting with the neuroregulatory component of the conditioned media, the secretome of HUVECs showed markedly decreased amounts of growth factors associated with neuronal growth and survival, including BDNF, GDNF, β-NGF, IGF-1, or S100-B (Figure 5A). This is the opposite to the other two conditions (ASCs and ASCs + HUVECs) where these neuroregulatory factors were upregulated. In fact, the highest relative expression values for BDNF, GDNF, β-NGF IGF-1, and S100 B were seen in the co-culture (Figure 5A). Additionally, it was also observed high amounts of IL-6 and MCP-1 on the secretome of ASCs and ASCs + HUVECs, two cytokines that have an important role in immune response following neuronal trauma (Supplementary Figure 1A). The analysis of angiogenic components followed the same trend, with the secretome of HUVECs in general presenting decreased expression of the factors, excepting EGF which presented increased relative amounts in the secretome of the ECs. These include TIMP-1 and TIMP-2, being the latter increased in the secretome of the co-culture, and CXCL-1/2/3 which was upregulated in the conditioned media of ASCs. The presence of Angiogenin and CXCL-5 was only observed whenever ASCs were present, showing both relative higher quantities in the secretome of ASCs + HUVECs (Figure 5B). Additionally, we also found similar amounts of bFGF, VEGF-A, and VEGF-D in the secretome of ASCs and ASCs + HUVECs (Supplementary Figure 1B).

Altogether, these results help to shed some insight in the way the secretome dynamics of ASCs might be affected by the presence of other cells on the environment. Thus, we observed a positive impact on the expression of neurotrophic factors when HUVECs were in culture together with ASCs and a very similar angiogenic profile for the secretome of ASCs and ASCs + HUVECs, a fact that may indicate that this modulation might be specific for some components of the secretome.

### Vascular Recruitment Is Potentiated by the Presence of ASCs

**FIGURE 6:**
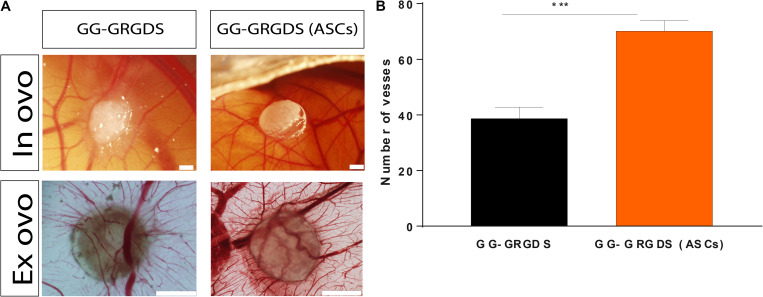
Vessel recruitment capacity of ASCs encapsulated in GG-GRGDS following 3 days of implantation on CAM. **(A)** Representative *in ovo* and *ex ovo* images of each condition. **(B)** Quantification of the number of vessels converging to the hydrogels demonstrated that these ASCs significantly impacted this parameter in comparison to the hydrogel without cells, which shows how these cells may affect the SCI vascular milieu following implantation. Scale bar: 1 mm. Results are shown as mean ± SEM (*n* = 12/14); ***p < 0.001.

To understand the capacity of GRGDS-modified GG encapsulating ASCs in recruiting blood vessels and induce vascular reorganization these were tested against the biomaterial without cells in an *in vivo* setting: the CAM assay. This model takes advantage of the highly vascularized CAM (grows by day 7 of chick embryonic development and matures by day 12) to study angiogenesis. It is a cheap and relatively quick way to study angiogenesis, especially for drug screening and implantation of biomaterial-based therapies aiming to transplant cells (due to limited immune responses, which allow the transplant of xenografts) serving as proof-of-concept before evolving to more complex *in vivo* models (Nowak-Sliwinska et al., 2014). Following 3 days of implantation (Figure 6A), the combination of hydrogel and ASCs exerted a chemoattractant effect on blood vessels as it significantly increased their convergence (70.17 ± 3.76 blood) when comparing to GRGDS alone (38.71 ± 4.09 blood vessels; Figure 6B). As previously detailed, this recruitment is likely to be mediated by the secretome of ASCs due to its highly rich angiogenic content. Therefore, these results show that ASCs have the potential to redesign the vascular milieu of SCI by promoting vascular attraction and reorganization of spared blood vessels (helping to revascularize the lesion site and prevent damage associated to vascular damage in SCI).

## Discussion

Vascular damage leading to BSCB disruption occurs in spinal cord trauma or laceration, playing a critical role in defining the severity of SCI. In fact, Noble and Wrathall (Noble and Wrathall, 1989a,b) demonstrated that the extension of the cystic cavity was similar to the intraparenchymal hemorrhage originated by vessel destruction and opened up the possibility that controlling this phenomena could lead to attenuate the severity of the condition. Even though the permeability of the BSCB is restored 14 days following injury, it is important to highlight that most vessels are not associated in neurovascular units, which are crucial in regulating appropriate blood flow to the spinal cord (Casella et al., 2002; Goritz et al., 2011).

Li et al. (2017) recently demonstrated that spinal cord tissue caudal to the lesion site is in permanent hypoxia. By artificially elevating O_2_ levels, the authors observed a prolonged increased on tissue oxygenation (in opposition to rostral to lesion where the values got back to normal 1 min after the stimulus) probably due to triggering neurovascular coupling, which further dilates vessels and increases oxygen and neuronal activity, originating transient locomotor gains of function (Li et al., 2017). This elegant study clearly underlines the importance of appropriate vascular functional for neuronal homeostasis.

Aiming to reshape the vascular milieu following SCI, in this work we started by understanding the potential of ASCs in modulating this parameter and how it would affect axonal growth. Thus, these MSCs were encapsulated with HUVECs in an in-house biomaterial-based approach (GG-GRGDS hydrogels) and the neurite outgrowth experiments showed that the co-culture provided potential for axonal regeneration similar to ASCs alone and superior to HUVECs or the hydrogel, opening the possibility of positive between both types of cells in the injury environment. In fact, the co-culture (as ASCs alone) proved to impact the internal neuronal growth machinery from early timepoints (12 h at least) as shown by the upregulation of a protein directly connected to axonal growth (GAP-43). This protein is highly expressed in regenerating axons where it acts to potentiate filopodia formation in growth cones (if phosphorylated) or induces microtubule-based outgrowth (if unphosphorylated; He et al., 1997; Dent and Meiri, 1998). One of the molecules that has been shown to upregulate GAP-43 is BDNF (Segal et al., 1995; Gupta et al., 2009), being essential for the neurotrophic action of the latter. Therefore, this is one of the mechanisms we propose for the neurite outgrowth results here reported.

The beneficial effects of ASCs on the organization of HUVECs were quite visible within the hydrogels, since these ECs were only capable of organizing in vascular-like structures when cultured in the presence of the MSCs. The characterization of the secretome of ASCs by our (Pires et al., 2016) and other groups (Nakagami et al., 2005; Kachgal and Putnam, 2011; Nakanishi et al., 2011) has showed an enrichment in angiogenic growth factors which could explain the impact of these MSCs on the vascular organization of HUVECs. Nevertheless, in line with our findings, others reported increased tube-formation capacity and vessel network stability of ECs when co-cultured in the presence of ASCs or other types of MSCs (Merfeld-Clauss et al., 2010; Holnthoner et al., 2015). These studies showed that ASCs induced an increased expression of CD31 in ECs and acted on VEGF, HGF and PDGF-BB pathways being their presence imperative for the formation and stabilization of such networks.

The present work also showed that the effect of ASCs on HUVECs probably created a positive synergy that can upregulate a different variety of neurotrophic factors. These molecules belong to the PI3K-Akt pathway, which our group previously identified as one of the effectors of the secretome of ASCs in injured spinal cord (Gomes et al., 2018). This pathway contributes to cell growth, proliferation and nutrient uptake and its activation has been shown to promote the regeneration of corticospinal tract neurons (Liu et al., 2010), and optic nerve axons (Park et al., 2008), making it a central signaling cascade to drive regeneration.

Moving away from classical neurotrophic factors, we desired to highlight the prevalence of IL-6, and MCP-1 in the secretome of ASCs and ASCs + HUVECs which are classically defined as mediators of the inflammatory response and can also impact neuronal regeneration (Leibinger et al., 2013; Kwon et al., 2015; Willis et al., 2020). Willis et al. (2020) showed that IL-6 induces neuroprotection in neurons following traumatic brain injury by a *trans-*signaling mechanism. MCP-1 seems to mediate the crosstalk between DRG neurons and macrophages, inducing neurite outgrowth, and mobilizing M2 macrophages (Kwon et al., 2015).

The analysis of angiotrophic molecules revealed the presence of several angiotrophic factors such as Angiogenin, EGF, bFGF, PDGF-BB, TPO as well as VEGF-A, and VEGF-D in the secretome of ASCs and ASCs+HUVECs. During these experiments it was clear that the secretome of HUVECs showed a clear downregulation, in some cases lack of expression of these factors, a fact that highlights the importance of adding ASCs to provoke their normal homeostasis in GG-GRGDS and might help to explain their incapacity to assembly into vascular-like networks in the absence of the MSCs. Two type of VEGF isoforms (VEGF-A and -D), a key family of angiogenic proteins, were detected on the analyzed secretomes. VEGF-A binds VEGFR1 and VEGFR2 and is crucial for vascular development during embryogenesis, continuing to stimulate angiogenesis postnatally, and having also a role in pathological angiogenesis (Holmes and Zachary, 2005). In addition this isoform is capable exerting neurotrophic and neuroprotective effects (trough flk-1), being a molecule capable of connecting angiogenesis with neuronal development (Sondell et al., 2000).

We also found increased levels of TIMP-1 and TIMP-2 in all the conditioned media collected from ASCs and ASCs+HUVECs collected in GG-GRGDS hydrogels, a possible reflection of the cellular interactions at the time of recollection. These were collected after 7 days of culture when the cells colonized the entire hydrogel and vascular networks (for the co-culture) were established leading to the inhibition of cellular migration, and the stabilization of the vascular networks formed.

CXCL5 and CXCL1/2/3 also showed increased relative amounts in the secretomes of ASCs + HUVECs and ASCs, respectively. Both chemokines impact angiogenesis after binding to CXCR2 (Mehrad et al., 2007; Chen et al., 2019). CXCL5 exerts angiogenic effects through the induction of VEGF-A by binding FOXD1 protein to a promoter of the growth factor (Chen et al., 2019). CXCL1/2/3 through CXCR2, leads to the activation and migration of ECs (Mehrad et al., 2007). These results allowed us to understand the relative expression of a myriad of angiogenic and neurotrophic effectors present in the secretome of ASCs and how it can be modulated by the presence of other cells in a 3D environment (in this case HUVECs).

Otherwise a bionert polymer, previous works have demonstrated that the insertion of RGD motifs into the backbone of GG allows the polymer to activate cellular integrins which in turn induces cellular adhesion, proliferation, survival, and regular homeostasis (Silva et al., 2012; Gomes et al., 2016). Accordingly, this chemical modification also impacts the quality of the secretome of MSCs since secretome collected from MSCs on GRGDS-modified GG enhanced neuronal survival, their proliferation and metabolism in comparison to native GG (Silva et al., 2013). Apart from these chemical cues, the physical properties of the three dimensional environment provided by hydrogels also have a profound impact on cellular behavior (Lee and Kim, 2018). Chaudhuri et al. (2016) designed RGD-modified alginate hydrogels with different stress relaxation properties and elegantly showed how the simple modulation of this physical parameter directed MSC differentiation and impacted cellular proliferation independently of RGD density. Taking this example into consideration, the elucidation on how such parameters govern the capacity of GRGDS-GG in promoting for instance the assembling HUVECs into vascular-like structures or the impact the physical properties of the hydrogel have on the secretome of ASCs should be explored in future studies.

Even though our *in vitro* results showed the potential ASCs have to influence angiogenesis and vascular organization of transplanted ECs, these still needed to be validated in an *in vivo-like* setting. The CAM assay enabled to observe that ASCs encapsulated in GG-GRGDS had a significantly higher capacity in recruiting blood vessels toward the hydrogel when compared to GG-GRGDS, a feature that shows the impact of their secretome on reshaping vascular organization and recruitment. This feature is of primordial importance when thinking on the implantation of a vascularization strategy in an animal model of SCI. Therefore, it shows that it might have the capacity to stimulate angiogenesis and with this induce the revascularization of the lesion site and possibly control the infiltration of inflammatory cells from the acute phase of the condition. Finally, this might allow to modulate the severity of the condition.

## Conclusion

In this work we started by validating *in vitro* the potential of developing a therapeutic approach aiming to restore normal homeostatic vasculature following SCI and with this to positively modulate inflammation with the final goal of impacting disease severity and motor and autonomic recovery. The co-culture ASCs + HUVECs in GG-GRGDS hydrogels lead to a similar neurite outgrowth and arborization in DRG explants as ASCs alone, impacting genetic expression of proteins involved in regeneration as early as 12 h following culture. Moreover, ASCs were pivotal for the arrangement of HUVECs into vascular-like structures a feature probably due not only to their paracrine action but also to cell-to-cell communication. The neurotrophic part of the secretome of ASCs and HUVECs showed an upregulation in some of these molecules, proving that the interaction between these MSCs and ECs may induce benefits to SCI environment. Regarding the angiotrophic molecules analyzed, the profile of ASCs and co-culture was similar, which helps to prove that the major changes in vascular assembly are majorly mediated by cell-cell contact. Finally, implantation of the hydrogel together with ASCs in the CAM lead to increased recruitment of blood vessels which shows the potential of these cell in reshaping the vascular milieu *in vivo*. Altogether, these results open up a promising possibility of implanting this biomaterial-based cell therapy in a SCI animal to study its impact on revascularization and functional recovery.

## Data Availability Statement

The raw data supporting the conclusions of this article will be made available by the authors, without undue reservation, to any qualified researcher.

## Ethics Statement

The studies involving human participants were reviewed and approved by LaCell LLC; Ethical Commission for Health of Braga Hospital. The patients/participants provided their written informed consent to participate in this study.

## Author Contributions

LR conducted the experiments, analyzed data, and wrote the manuscript. EG, JA, and SG helped during the cell culture and CAM experiments, contributing to data analysis. FB, NS, RS, and DL helped to draft the manuscript and revised it critically. AS conceived the analysis, participated in its design and coordination, helped to draft the manuscript, and gave the final approval of the version to be published. All authors read and approved the final manuscript.

## Conflict of Interest

The authors declare that the research was conducted in the absence of any commercial or financial relationships that could be construed as a potential conflict of interest.
