# Supplementary figures and images for: In vitro Evaluation of ASCs and HUVECs Co-cultures in 3D Biodegradable Hydrogels on Neurite Outgrowth and Vascular Organization
Source: Front Cell Dev Biol. 2020 Jun 16;8:489. doi: 10.3389/fcell.2020.00489 (PMC7308435; doi:10.3389/fcell.2020.00489)

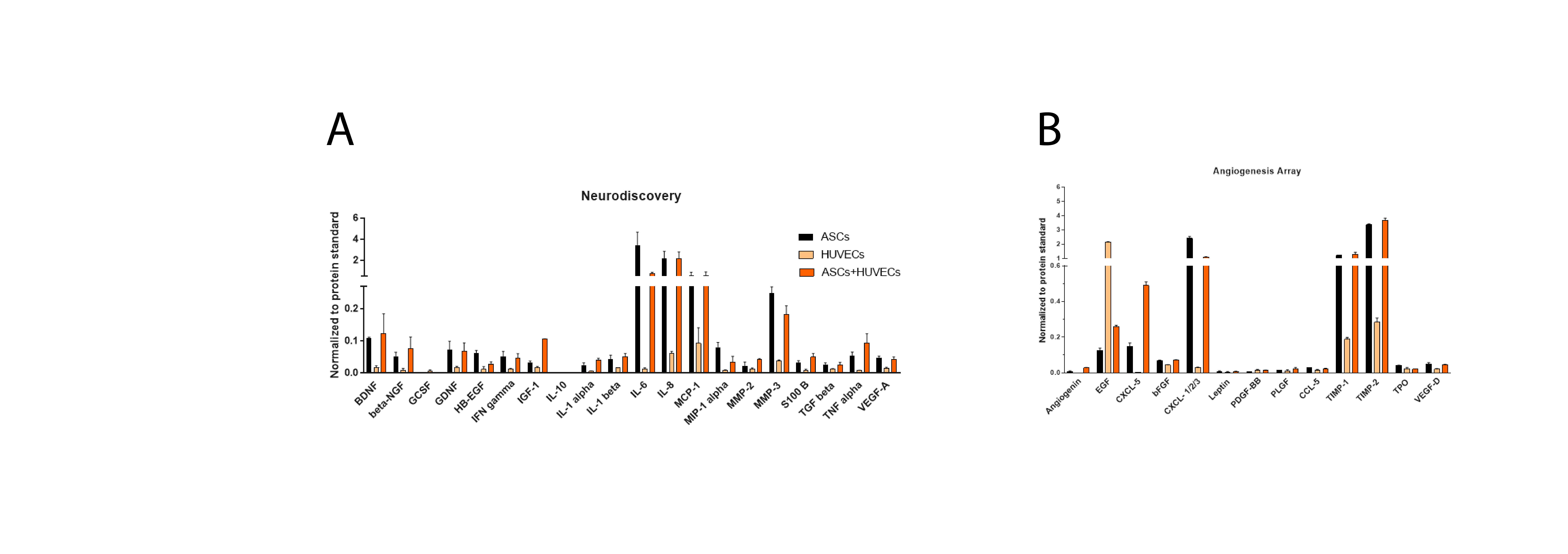

Supplement: Supplementary file 1 [file Image_1.tif]
